# High-yield production of multiple *O*-methylated phenylpropanoids by the engineered *Escherichia coli*–*Streptomyces* cocultivation system

**DOI:** 10.1186/s12934-019-1118-9

**Published:** 2019-04-10

**Authors:** Heqing Cui, Myoung Chong Song, Yeon Hee Ban, Soo Youn Jun, An Sung Kwon, Ji Young Lee, Yeo Joon Yoon

**Affiliations:** 1 Department of Chemistry and Nanoscience, Ewha Womans University, Ewhayeodae-gil 52, Seoul, 03760 Republic of Korea; 2 iNtRON Biotechnology, Inc., Seongnam-si, Gyeonggi-do 13202 Republic of Korea

**Keywords:** *O*-methylated phenylpropanoids, *O*-methyltransferase, *E. coli*–*Streptomyces* cocultivation

## Abstract

**Background:**

*O*-Methylated phenylpropanoids, which are generally present in small amounts in plants, have improved or distinct biological activities and pharmacological properties as opposed to their unmethylated counterparts. Although microbial production could be a useful tool for the efficient and environment-friendly production of methylated phenylpropanoids, a high-yield microbial production of neither tri-methylated stilbenes nor di-/tri-methylated flavonoids has been achieved to date.

**Results:**

A methyltransferase from *Streptomyces avermitilis* (SaOMT2), which has been known to possess 7-*O*-methylation activity toward several flavonoids, exhibited more diverse regiospecificity and catalyzed mono-, di-, and tri-methylation of stilbene, flavanone, and flavone when it was expressed in *Streptomyces venezuelae*. For the efficient production of multi-methylated phenylpropanoids, a cocultivation system was developed by employing engineered *Escherichia coli* strains producing pterostilbene, naringenin, and apigenin, respectively, along with SaOMT2-expressing *S. venezuelae* mutant. Consequently, high-yield microbial production of tri-methylated stilbenes and di-/tri-methylated flavonoids (including 3,5,4′-trimethoxystilbene, 5-hydroxy-7,4′-dimethoxyflavanone, 4′-hydroxy-5,7-dimethoxyflavanone, 5,7,4′-trimethoxyflavanone, 5-hydroxy-7,4′-dimethoxyflavone, and 5,7,4′-trimethoxyflavone) has been demonstrated for the first time.

**Conclusions:**

This cocultivation system based on the phenylpropanoid-producing *E. coli* and SaOMT2-expressing *S. venezuelae* provides an efficient tool for producing scarce and potentially valuable multi-methylated phenylpropanoids and will enable further development of these compounds as pharmaceuticals and nutraceuticals.

**Electronic supplementary material:**

The online version of this article (10.1186/s12934-019-1118-9) contains supplementary material, which is available to authorized users.

## Background

**Fig. 1:**
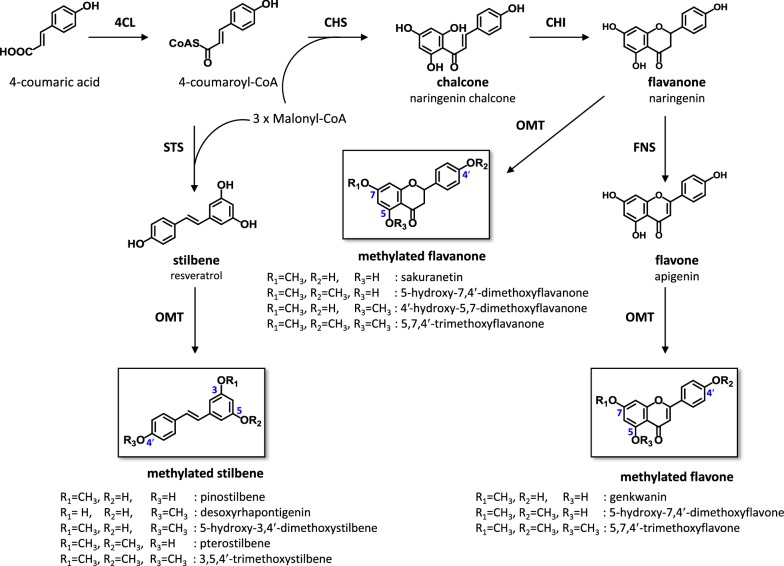
Schematic of the biosynthetic pathway from 4-coumaric acid to flavonoid and stilbene. 4CL, 4-coumarate:CoA ligase; CHS, chalcone synthase; STS, stilbene synthase; CHI, chalcone isomerase; FNS, flavone synthase; OMT, *O*-methyltransferase

Phenylpropanoids, including flavonoids and stilbenes, are structurally diverse plant secondary metabolites that have significant potential as pharmaceuticals, nutraceuticals, and cosmetics owing to their antioxidation, cancer prevention, anticancer, antibacterial, and anti-inflammatory properties [1]. However, their availability from plants is limited owing to seasonal or regional variations, low abundance, and the difficulty in isolating single compounds from complex mixtures. Therefore, microbial production of these important metabolites by expressing plant biosynthetic genes in microbial hosts is an efficient alternative for large-scale and environment-friendly production [2]. The biosynthesis of the parent skeleton of flavonoids begins with the conversion of 4-coumaric acid, derived from phenylalanine or tyrosine, to 4-coumaroyl-CoA by 4-coumarate:CoA ligase (4CL). The subsequent condensation of 4-coumaroyl-CoA with three malonyl-CoA by chalcone synthase (CHS) produces naringenin chalcone, which is converted to simple flavanone naringenin by chalcone isomerase (CHI). The stilbene synthase (STS) acts on 4-coumaroyl-CoA and catalyzes sequential condensation, resulting in the production of resveratrol (one of the simplest stilbenes). The simple phenylpropanoid skeletons are further modified by various enzymes such as flavone synthase (FNS) and flavonol synthase as well as by diverse methylation and glycosylation (Fig. 1) [2].

It has been reported that methylated flavonoids and stilbenes exhibit significantly higher metabolic stability, oral bioavailability, and biological activity than their unmethylated counterparts [3]. For example, pterostilbene (4′-hydroxy-3,5-dimethoxystilbene) (Fig. 1) which is a dimethylated analog of resveratrol, exhibited a dramatically higher bioavailability [4] and longer half-life than resveratrol [5]. However, it has been known that 100 g of blueberries contains only 15 μg of pterostilbene [6]. In case of flavonoids, sakuranetin (5,4′-dihydroxy-7-methoxyflavanone) (Fig. 1) exhibited antifungal activity against *Pyricularia oryzae*, but only 10 mg of sakuranetin can be isolated from 50 g of ultraviolet-irradiated rice leaves [7]. Tri-methylated resveratrol (3,5,4′-trimethoxystilbene) (Fig. 1), which can be found from several plants but only in small amounts [8], has been chemically synthesized and found to show more potent anticancer and antiangiogenic activities than resveratrol [9, 10]. Furthermore, tri-methylated naringenin (5,7,4′-trimethoxyflavanone) (Fig. 1) was isolated from a complex flavonoid mixture extracted from plants including *Aglaia duperreana* and *Praxelis clematidea* R.M. King & Robinson; this naringenin trimethyl ether exhibited considerable molluscicidal activity against golden apple snail, an agricultural pest [11], antibacterial activity against Gram-positive and Gram-negative bacteria [12], and antifungal activity against *Candida* species [13]. Fortunately, phenylpropanoids and their methylated analogs can be produced from genetically engineered microbial hosts. For example, approximately 35 mg/L of pterostilbene was produced in *Saccharomyces cerevisiae* by expressing resveratrol biosynthetic genes and *O*-methyltransferase (OMT) gene from *Vitis vinifera* (VvROMT) and carrying out further optimization [14]. Expression of naringenin biosynthesis pathway along with 7-OMT from *Oryza sativa* L. cv. Nakdong in *Escherichia coli* produced approximately 40 mg/L of sakuranetin (Fig. 1) [15]. Genkwanin (5,4′-dihydroxy-7-methoxyflavone; Fig. 1) was also produced in *E. coli* at a yield of 41 mg/L by adopting a similar approach using 7-OMT of poplar (POMT7) [16]. However, the microbial production of tri-methylated phenylpropanoids has rarely been reported. Only trace amounts (~ 0.2 mg/L) of 3,5,4′-trimethoxystilbene (Fig. 1) was produced by expressing resveratrol biosynthetic genes together with two OMT-encoding codon-optimized genes from *Sorghum bicolor* in *E. coli* [17]. Notably, until recently, the microbial production of neither di- nor tri-methylated flavonoids had been reported.

Here, we recharacterized the function of 7-OMT from *Streptomyces avermitilis* ATCC 31267 (SaOMT2). SaOMT2 expressed in *E. coli* reportedly exhibits 7-*O*-methylation activity toward naringenin as well as some isoflavones and flavones [18]. However, we found that SaOMT2 expressed in *Streptomyces venezuelae* was able to di- and tri-methylate resveratrol, naringenin, and apigenin. Furthermore, we developed a cocultivation system employing each of the engineered *E. coli* strains that produced pterostilbene, naringenin, and apigenin, respectively, along with SaOMT2-expressing *S. venezuelae* mutant for achieving a high-yield production of multi-methylated stilbene, flavanone, and flavone. Optimization of cocultivation conditions resulted in the production of approximately 29 mg/L of 3,5,4′-trimethoxystilbene, which is the highest titer reported to date. To our knowledge, the production of a series of di- or tri-methylated naringenin and apigenin analogs based on a cocultivation system is the first report concerning microbial production of multi-methylated flavonoid compounds. This one-pot cocultivation system provides a facile tool for the high-yield production of such scarce and potentially valuable methylated flavonoids and stilbenes and will enable further development of these compounds.

## Results

### In vivo characterization of *S. avermitilis* OMT expressed in *S. venezuelae*

**Fig. 2:**
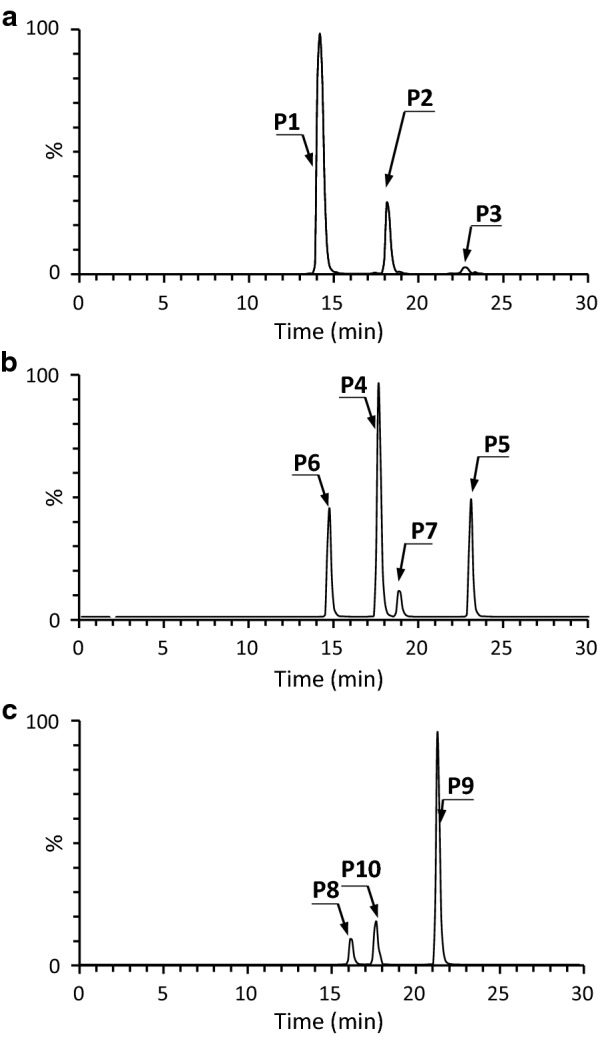
UPLC–qTOF–HR-MS analyses of methylated stilbene, flavanone, and flavone produced by bioconversion using DHS2001/SaOMT2. **a** UPLC–qTOF–HR-MS chromatogram selected for *m/z *= 243.1016, 257.1172, and 271.1329 corresponding to desoxyrhapontigenin (**P1**), 5-hydroxy-3,4′-dimethoxystilbene (**P2**), and 3,5,4′-trimethoxystilbene (**P3**), respectively, of culture extracts obtained from DHS2001/SaOMT2 supplemented with resveratrol. **b** UPLC–qTOF–HR-MS chromatogram selected for *m/z *= 287.0914 corresponding to sakuranetin (**P4**), *m/z *= 301.1071 corresponding to 5-hydroxy-7,4′-dimethoxyflavanone (**P5**) and 4′-hydroxy-5,7-dimethoxyflavanone (**P6**), and *m/z *= 315.1227 corresponding to 5,7,4′-trimethoxyflavanone (**P7**) of culture extracts obtained from DHS2001/SaOMT2 supplemented with naringenin. **c** UPLC–qTOF–HR-MS chromatogram selected for *m/z *= 285.0757, 299.0914, and 313.1071 corresponding to genkwanin (**P8**), 5-hydroxy-7,4′-dimethoxyflavone (**P9**), and 5,7,4′-trimethoxyflavone (**P10**), respectively, of culture extracts obtained from DHS2001/SaOMT2 supplemented with apigenin

It has been reported that the *E. coli* strain expressing one of the OMT from *S. avermitilis* ATCC 31267 (SaOMT2; GenBank accession no. BAC70093) can catalyze 7-*O*-methylation of flavanone (naringenin), isoflavone (daidzein and genistein), and flavone (apigenin, quercetin, kaempferol, and isorhamnetin) [18]. Nevertheless, because SaOMT2 is originated from *Streptomyces* species, we opted to re-examine the regiospecificity of SaOMT2 expressed in a *Streptomyces* host. In our previous study, *S. venezuelae* DHS2001, in which the native pikromycin polyketide synthase gene was deleted, has been successfully used to produce flavanones and stilbenes by heterologous expression of codon-optimized genes derived from various plants [19]. To construct the DHS2001/SaOMT2 mutant strain, SaOMT2 was expressed in *S. venezuelae* DHS2001 using a replicative *E. coli*–*Streptomyces* shuttle vector pSE34 [20] carrying the strong constitutive ermE* promoter [21]. Resveratrol, naringenin, and apigenin were fed to a culture of 48 h-precultivated DHS2001/SaOMT2 at a concentration of 100 mM and subsequently incubated for 40 h. All the supplemented substrates were completely converted to their corresponding mono-, di-, and tri-methylated compounds. Organic extracts of DHS2001/SaOMT2 fed with resveratrol were analyzed by ultra performance liquid chromatography–quadrupole time-of-flight high resolution mass spectrometry (UPLC–qTOF–HR-MS), and the peaks corresponding to desoxyrhapontigenin (3,5-dihydroxy-4′-methoxystilbene), 5-hydroxy-3,4′-dimethoxystilbene, and 3,5,4′-trimethoxystilbene were observed at conversion yields of approximately 75.8%, 20.3%, and 3.9%, respectively (see Fig. 1 for their structures; Fig. 2a). The identities of these methylated resveratrol analogs were verified by comparing their UPLC retention times and HR-MS/MS fragmentation patterns with those of the commercial authentic standards (Additional file 1: Figure S1). The UPLC–qTOF–HR-MS analysis of the organic extract obtained from the DHS2001/SaOMT2 fed with naringenin produced UPLC peaks predicted to correspond to sakuranetin, 5-hydroxy-7,4′-dimethoxyflavanone, 4′-hydroxy-5,7-dimethoxyflavanone, and 5,7,4′-trimethoxyflavanone (Figs. 1, 2b) at approximately 46.9%, 23.1%, 24%, and 6% yields, respectively. The structures of sakuranetin, 5-hydroxy-7,4′-dimethoxyflavanone, 4′-hydroxy-5,7-dimethoxyflavanone, and 5,7,4′-trimethoxyflavanone were confirmed based on their retention times and the patterns produced in MS/MS analysis after comparing with those of authentic standards (Additional file 1: Figure S2). The UPLC–qTOF–HR-MS analysis of the extract of DHS2001/SaOMT2 fed with apigenin showed peaks corresponding to genkwanin, 5-hydroxy-7,4′-dimethoxyflavone, and 5,7,4′-trimethoxyflavone. The conversion yields for each product were 8%, 76.7%, and 15.3%, respectively (Figs. 1, 2c). Their identities were again confirmed by UPLC and MS/MS analysis in comparison with the authentic standards (Additional file 1: Figure S3). The same experiments were performed using DHS2001, but no methylated product was detected in UPLC–qTOF–HR-MS analysis. These results demonstrate that, when SaOMT2 derived from *S. avermitilis* was expressed in the *Streptomyces* heterologous host, SaOMT2 not only shows mono-methylation activity on 7-hydroxy group of flavanone, isoflavone, and flavone but also di- and tri-methylation activity on the positions 3, 5, and 4′ of stilbene and the positions 5, 7, and 4′ of flavanone/flavone.

### In vitro characterization of *S. avermitilis* OMT expressed in *E. coli* and *S. venezuelae*

**Fig. 3:**
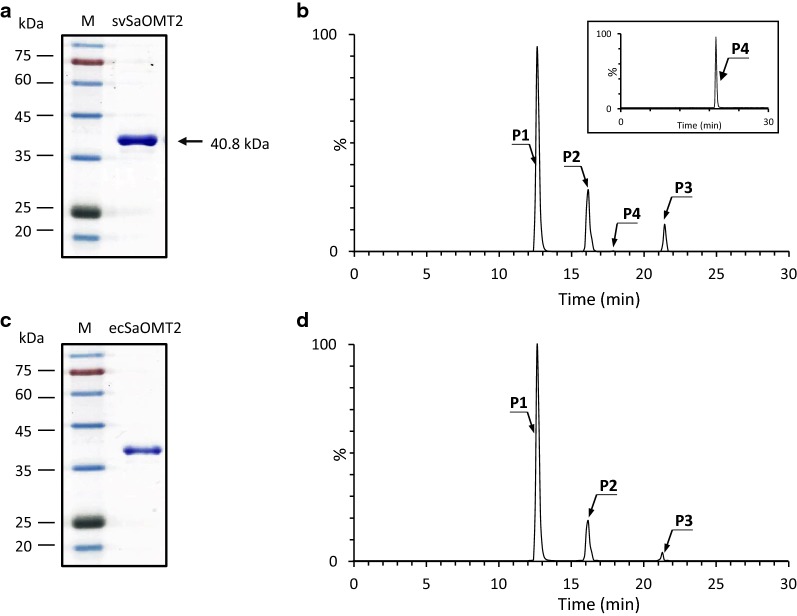
Analysis of in vitro methylation reaction catalyzed by recombinant SaOMT2. **a** SDS-PAGE of purified SaOMT2 expressed in *S. venezuelae* (svSaOMT2). Lane M shows the molecular weight marker with the indicated masses. **b** UPLC–qTOF–HR-MS chromatogram selected for *m/z *= 285.0757, 299.0914, and 313.1071 corresponding to genkwanin (**P2**), 5-hydroxy-7,4′-dimethoxyflavone (**P3**), and 5,7,4′-trimethoxyflavone (**P4**), respectively, converted from apigenin (**P1**) by SaOMT2 expressed in *S. venezuelae*. **c** SDS-PAGE of purified SaOMT2 expressed in *E. coli* (ecSaOMT2). **d** UPLC– qTOF–HR-MS chromatogram selected for *m/z *= 285.0757 and 299.0914 corresponding to genkwanin (**P2**) and 5-hydroxy-7,4′-dimethoxyflavone (**P3**), respectively, converted from apigenin (**P1**) by SaOMT2 expressed in *E. coli*

To examine the multi-methylation activity of SaOMT2 in vitro, we expressed the histidine-tagged recombinant SaOMT2 in both *S. venezuelae* and *E. coli* and subjected it to purification (Fig. 3a, c). When the purified SaOMT2 expressed in *S. venezuelae* was incubated overnight with apigenin in the presence of *S*-adenosyl-l-methionine (SAM) as a methyl donor, the products corresponding to genkwanin, 5-hydroxy-7,4′-dimethoxyflavone, and 5,7,4′-trimethoxyflavone were produced at conversion yields of approximately 26%, 10%, and 0.1%, respectively (Fig. 3b). In contrast, incubation of *E. coli*-expressed SaOMT2, whose codons were optimized for facilitating efficient expression in *E. coli,* with apigenin and SAM under the same conditions resulted in the production of UPLC peaks corresponding to genkwanin and 5-hydroxy-7,4′-dimethoxyflavone at 19% and 3% yields, respectively (Fig. 3d). These results confirmed that *S. venezuelae*-expressed SaOMT2 possesses mono-, di-, and tri-methylation activity although the conversion yield to tri-methylated 5,7,4′-trimethoxyflavone was low in vitro, whereas *E. coli*-expressed SaOMT2 exhibits only mono- and di-methylation activities toward flavone. Increasing the enzyme amount and incubation period in the experiments using *E. coli*-expressed SaOMT2 did not produce 5,7,4′-trimethoxyflavone. Furthermore, *S. venezuelae*-expressed SaOMT2 exhibited remarkably higher mono- and di-methylation activities than *E. coli*-expressed SaOMT2. Interestingly, di-methylation activity of *E. coli*-expressed SaOMT2 has not been identified in the previous report in which the whole cell *E. coli* expressing SaOMT2 was used as a biocatalyst [18].

### Development and optimization of the cocultivation system

**Fig. 4:**
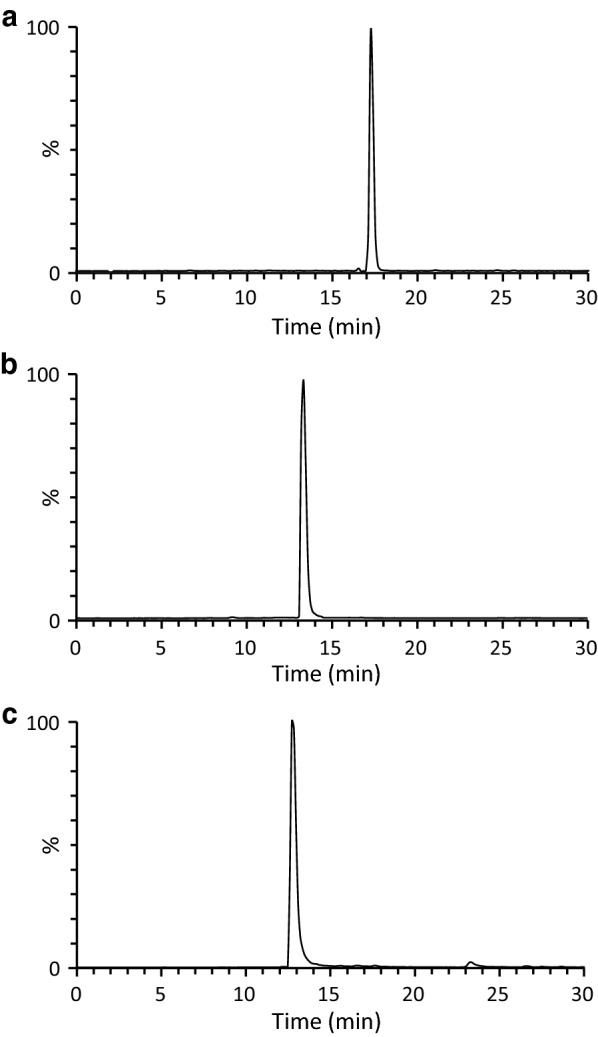
UPLC–qTOF–HR-MS analyses of pterostilbene, flavanone, and flavone produced by phenylpropanoid-producing *E. coli* mutants. **a** UPLC–qTOF–HR-MS chromatogram of pterostilbene (*m/z *= 257.1172) obtained from BL21/PTS supplemented with 4-coumaric acid. **b** UPLC–qTOF–HR-MS chromatogram of naringenin (*m/z *= 273.0757) obtained from BL21/NRG supplemented with 4-coumaric acid. **c** UPLC–qTOF–HR-MS chromatogram of apigenin (*m/z *= 271.0601) obtained from BL21/APG supplemented with 4-coumaric acid

The discovery of stronger and more diverse regiospecific methylation activity of *S. venezuelae*-expressed SaOMT2 in comparison with that of *E. coli*-expressed SaOMT2 motivated us to develop an *E. coli*–*Streptomyces* cocultivation system for the efficient microbial production of multi-methylated flavonoids and stilbenes because *E. coli* is a more favorable heterologous host for the production of simple phenylpropanoid backbone in terms of high titer, fast growth, and ease of genetic manipulation [2]. First, we engineered an *E. coli* strain producing a di-methylated stilbene, pterostilbene, rather than resveratrol using 4-coumaric acid as the substrate because the di- and tri-methylation activity of *S. venezuelae*-expressed SaOMT2 toward resveratrol is relatively weaker compared with that toward flavanone and flavone (Fig. 2); however, another OMT from *V. vinifera* (VvROMT; GenBank accession no. FM178870) is known to efficiently produce pterostilbene from resveratrol [22]. Naringenin- and apigenin-producing *E. coli* strains were also constructed. The genes for the synthesis of phenylpropanoid backbones were synthesized but were not codon-optimized because it had been reported that the plant genes could be successfully used for the production of desired compounds in *E. coli* [15, 16, 23]. The plasmid for pterostilbene synthesis, pPTS, was constructed using pET32a vector for expressing the synthetic 4CL gene from *O. sativa* (Os4CL; GenBank accession no. BAD27987) [23]; STS gene from *V. vinifera* (VvSTS; GenBank accession no. DQ459351) [24]; and OMT gene from *V. vinifera* (VvROMT) (Table 1). The plasmid, pNRG, was constructed using the pCDFduet-1 vector for inducing the biosynthesis of naringenin by expressing the synthesized Os4CL gene, CHS gene from *Populus euramericana* (PeCHS; TIGR accession no. TC29789) [25], and CHI gene from *Medicago truncatula* (MtCHI; GenBank accession no. XM_003592713) [16]. The ampicillin resistant gene was additionally inserted to the pCDFduet-1 vector as a selection marker during cocultivation with *S. venezuelae,* which is insensitive to ampicillin. The pAPG plasmid was designed for biosynthesizing apigenin by adding the synthetic FNS gene from *Petroselinum crispum* to pNRG (PcFNS; GenBank accession no. AY230247) [26]. These engineered plasmids were separately introduced into *E. coli* BL21(DE3) to generate BL21/PTS, BL21/NRG, and BL21/APG, respectively (Table 1). The engineered *E. coli* strains, whose gene expression was induced by isopropyl *β*-d-1-thiogalactopyranoside (IPTG), were grown on LB medium for 3 h at 37 °C and subsequently transferred to R2YE medium [27] supplemented with 1.2 mM (196.8 mg/L) of 4-coumaric acid and further incubated at 30 °C for 18 h; the latter is considered to be a favorable growth medium and temperature for *Streptomyces*. UPLC–qTOF–HR-MS analysis of organic extracts obtained from the engineered *E. coli* strains revealed peaks that were consistent with those of authentic pterostilbene, naringenin, and apigenin standards (Fig. 4; Additional file 1: Figure S4). Approximately, 79 mg/L, 134 mg/L, and 52 mg/L of pterostilbene, naringenin, and apigenin, respectively, were produced. These results validated that the engineered *E. coli* strains successfully produced and secreted the desired phenylpropanoid backbones when cultured under optimal growth conditions for *Streptomyces*, which allows further methylation by cocultivation with SaOMT2-expressing *S*. *venezuelae*.

**Table 1:** Plasmids and strains used in the present study

Plasmids/strains	Relevant characteristics	Source or reference
Plasmids		
pET32a	T7 promoter, f1 ori, Amp^r^	Novagen
pCDFduet-amp	double T7 promoter, CloDE13 ori, Str^r^, Amp^r^	This study [21]
pSE34	ermE* promoter, pIJ101 ori, Thio^r^	
pET-SaOMT2-H	pET32a carrying the SaOMT2 gene fused with a C-terminal histidine-tag	This study
pPTS	pET32a carrying Os4CL, VvSTS, and VvROMT genes	This study
pNRG	pCDFduet-amp carrying Os4CL, PeCHS, and MtCHI genes	This study
pNRG-SaOMT2	pCDFduet-amp carrying Os4CL, PeCHS, MtCHI, and SaOMT2 genes	This study
pAPG	pCDFduet-amp carrying Os4CL, PeCHS, MtCHI, and PcFNS genes	This study
pAPG-SaOMT2	pCDFduet-amp carrying Os4CL, PeCHS, MtCHI, PcFNS, and SaOMT2 genes	This study
pSE-SaOMT2	pSE34 carrying the SaOMT2 gene	This study
pSE-SaOMT2-H	pSE34 carrying the SaOMT2 gene fused with a C-terminal histidine-tag	This study
Strains		
*E. coli* BL21 (DE3)	F^−^ *ompT hsdS*_*B*_(r_B_^−^ m_B_^−^) *gal dcm lon* (DE3)	Novagen
DHS2001	*Streptomyces venezuelae* Δ pikromycin polyketide synthase	[33]
BL21/SaOMT2-H	*E. coli* BL21(DE3) strain harboring pET-SaOMT2-H	This study
BL21/PTS	*E. coli* BL21(DE3) strain harboring pPTS	This study
BL21/NRG	*E. coli* BL21(DE3) strain harboring pNRG	This study
BL21/NRG-SaOMT2	*E. coli* BL21(DE3) strain harboring pNRG-SaOMT2	This study
BL21/APG	*E. coli* BL21(DE3) strain harboring pAPG	This study
BL21/APG-SaOMT2	*E. coli* BL21(DE3) strain harboring pAPG-SaOMT2	This study
DHS2001/SaOMT2	DHS2001 strain harboring pSE-SaOMT2	This study
DHS2001/SaOMT2-H	DHS2001 strain harboring pSE-SaOMT2-H	This study

**Fig. 5:**
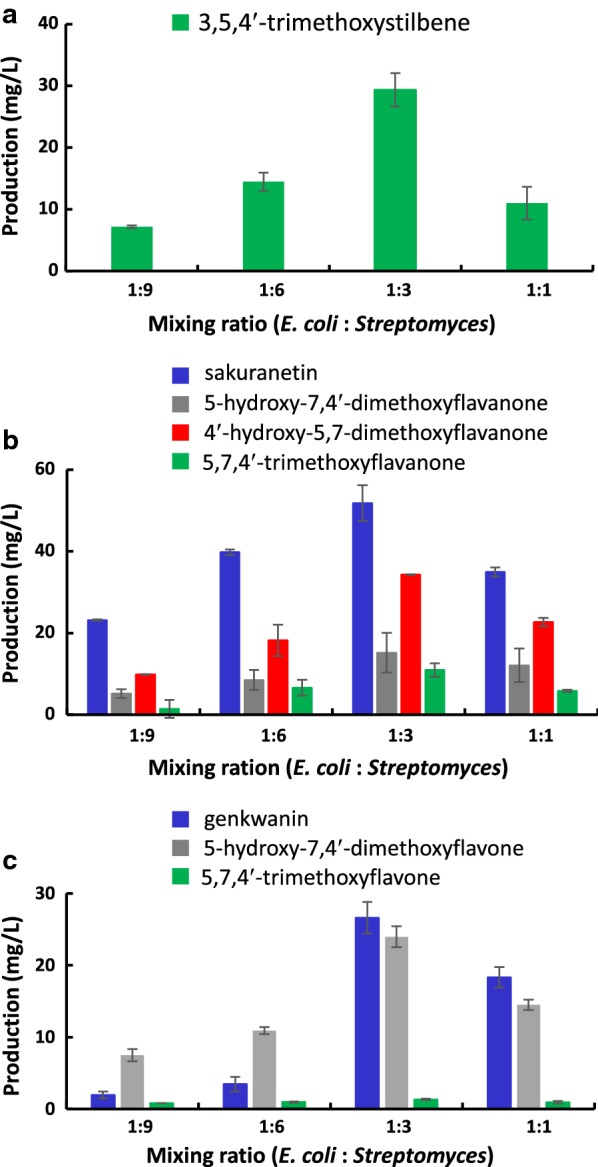
Production of methylated stilbene and flavonoid using different mixing ratios of *E. coli* and *Streptomyces* mutants. **a** The production of 3,5,4′-trimethoxystilbene by cocultivating BL21/PTS and DHS2001/SaOMT2. **b** The production of sakuranetin, 5-hydroxy-7,4′-dimethoxyflavanone, 4′-hydroxy-5,7-dimethoxyflavanone, and 5,7,4′-trimethoxyflavanone by cocultivating BL21/NRG and DHS2001/SaOMT2. **c** The production of genkwanin, 5-hydroxy-7,4′-dimethoxyflavone, and 5,7,4′-trimethoxyflavone by cocultivating BL21/APG and DHS2001/SaOMT2

**Fig. 6:**
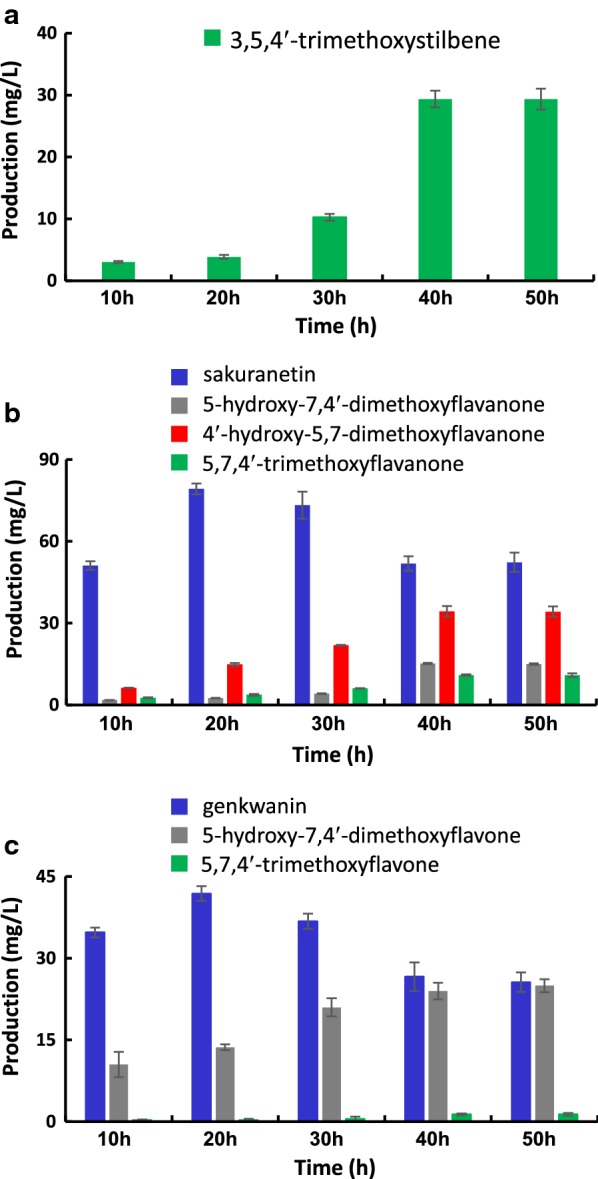
Production of methylated stilbene and flavonoid in the coculture system for a different cocultivation period. **a** The production of 3,5,4′-trimethoxystilbene by cocultivating BL21/PTS and DHS2001/SaOMT2. **b** The production of sakuranetin, 5-hydroxy-7,4′-dimethoxyflavanone, 4′-hydroxy-5,7-dimethoxyflavanone, and 5,7,4′-trimethoxyflavanone by cocultivating BL21/NRG and DHS2001/SaOMT2. **c** The production of genkwanin, 5-hydroxy-7,4′-dimethoxyflavone, and 5,7,4′-trimethoxyflavone by cocultivating BL21/APG and DHS2001/SaOMT2

*Streptomyces venezuelae* has a relatively faster growth rate (an approximate doubling time of 1 h) than that of other streptomycetes including *Streptomyces coelicolor* and *Streptomyces lividans*, which are widely-used as heterologous hosts [28]. However, in view of its significantly slower growth rate than that of *E. coli*, the precultivation period for phenylpropanoid-producing *E. coli* and SaOMT2-expressing *S. venezuelae* should be adjusted. We first decided to mix 3 h-old *E. coli* after induction and 40 h-old DHS2001/SaOMT2 (when it shows maximum cell growth) at the beginning of cocultivation. Next, to optimize the mixing ratio for *E. coli* and *S. venezuelae* mutants, the *E. coli* cells of BL21/PTS, BL21/NRG, or BL21/APG that were recovered by centrifugation were mixed with the recovered *S. venezuelae* cells of DHS2001/SaOMT2 grown on R2YE medium at varying weight ratios of 1:1, 1:3, 1:6, and 1:9 to make up a total volume of 15 mL in R2YE medium. The cocultures were fed with 1.2 mM of 4-coumaric acid and further incubated for 40 h at 30 °C. The production of 3,5,4′-trimethoxystilbene, which had undergone an additional *O*-methylation at the position 4′ of pterostilbene, from the coculture of BL21/PTS and DHS2001/SaOMT2 was analyzed by UPLC–qTOF–HR-MS. A cocultivation system in which *E. coli* and *S. venezuelae* were mixed at a ratio of 1:3 presented the highest yield of 3,5,4′-trimethoxystilbene (~ 29 mg/L) (Fig. 5a). Similarly, cocultivation of BL21/NRG or BL21/APG with DHS2001/SaOMT2 mixed at the ratio of 1:3 produced the highest amounts of mono-, di-, and tri-methylated compounds (Fig. 5b, c). Cocultivation of BL21/NRG with DHS2001/SaOMT2 for 40 h produced ~ 52 mg/L of sakuranetin, ~ 15 mg/L of 5-hydroxy-7,4′-dimethoxyflavanone, ~ 34 mg/L of 4′-hydroxy-5,7-dimethoxyflavanone, and ~ 11 mg/L of 5,7,4′-trimethoxyflavanone (Fig. 5b), while cocultivation of BL21/APG with DHS2001/SaOMT2 yielded ~ 27 mg/L of genkwanin, ~ 24 mg/L of 5-hydroxy-7,4′-dimethoxyflavone, and ~ 1 mg/L of 5,7,4′-trimethoxyflavone (Fig. 5c). Moreover, to determine the optimal cocultivation period, the production of methylated compounds of each cocultivation system was monitored for 10–50 h. The highest production of 3,5,4′-trimethoxystilbene (~ 29 mg/L) was observed with 40–50 h cocultivation of BL21/PTS with DHS2001/SaOMT2 (Fig. 6a). This is the highest tri-methylated resveratrol titer that has been reported to date. The highest production of sakuranetin was achieved after cocultivating BL21/NRG with DHS2001/SaOMT2 for 20–30 h, whereas the production of di- and tri-methylated flavanones was maximum after 40–50 h cocultivation. To the best of our knowledge, high yield productions of 5-hydroxy-7,4′-dimethoxyflavanone (~ 15 mg/L), 4′-hydroxy-5,7-dimethoxyflavanone (~ 34 mg/L), and 5,7,4′-trimethoxyflavanone (~ 11 mg/L) are the first examples of microbial multi-methylated flavanone production (Fig. 6b). The production of a mono-methylated flavone, genkwanin, was the highest at 20 h cocultivation of BL21/APG with DHS2001/SaOMT2, whereas the maximum production of 5-hydroxy-7,4′-dimethoxyflavone (~ 24 mg/L) and 5,7,4′-trimethoxyflavone (~ 1 mg/L) was observed after carrying out cocultivation for 40–50 h (Fig. 6c); to our knowledge, this finding is the first reported evidence for the microbial production of multi-methylated flavone.

Finally, to examine the possibility of direct production of multi-methylated flavonoids and stilbenes by *E. coli* strains, the BL21/NRG-SaOMT2 and BL21/APG-SaOMT2 strains were constructed by additionally expressing the synthetic codon-optimized SaOMT2 in BL21/NRG and BL21/APG. The resulting BL21/NRG-SaOMT2 and BL21/APG-SaOMT2 strains produced only small amounts of the respective mono-methylated compounds, sakuranetin and genkwanin (Additional file 1: Figure S5). These results are consistent with those of a previous study in which SaOMT2-expressing *E. coli* strain showed only mono-methylation activity toward flavanone and flavone compounds [18], and thus, this study demonstrates the advantage of *E. coli*–*Streptomyces* cocultivation system for efficient multi-methylation of flavonoids and stilbenes.

## Discussion

It has been known that the use of the whole cell *E. coli* expressing SaOMT2 as a biocatalyst can lead to mono-methylation at the position 7 of flavanone, isoflavone, and flavone [18]. However, we found that the purified SaOMT2 expressed in *E. coli* is able to mediate in vitro di-methylation at the positions 7 and 4′ of flavone (apigenin) as well as mono-methylation at the position 7 of the same compound (Fig. 3). The expression of this *S. avermitilis*-derived OMT in the same *Streptomyces* species *S. venezuelae* showed broader regiospecificity than that shown by *E. coli*-expressed SaOMT2. We observed that the SaOMT2-expressing *S. venezuelae* (DHS2001/SaOMT2) not only catalyzes 7,4′- and 5,7-di-methylation but also 5,7,4′-tri-methylation of flavanone (naringenin) and flavone (apigenin) by carrying out whole-cell bioconversion. Additionally, DHS2001/SaOMT2 mediates 3,5,4′-tri-methylation as well as 3,4′-di-methylation of stilbene (resveratrol) (Fig. 2). Moreover, the purified recombinant SaOMT2 obtained from *S. venezuelae* showed approximately 1.4- and 3.3-fold higher mono- and di-methylation activities toward apigenin, respectively, than those shown by SaOMT2 expressed in *E. coli* (Fig. 3). At present, it is unclear why SaOMT2 s expressed in *S. venezuelae* and *E. coli* have different activities and regiospecificities. However, it is plausible that heterologous expression of SaOMT2 in the same species facilitates more productive folding of this enzyme compared with its expression in different species.

OMTs that can catalyze the di-methylation reaction of stilbene and flavonoids have rarely been reported. For example, the OMT from *V. vinifera* (VvROMT), which we used for constructing pterostilbene-producing *E. coli* (BL21/PTS) in this study, and that from *S. bicolor* (SbOMT3) have 3,5-di-methylation activity toward resveratrol [17, 22]. Although tri-methylated stilbene, such as 3,5,4′-trimethoxystilbene, exists in the plant [8], the OMT(s) responsible for this multi-methylation reaction has not yet been characterized. Therefore, this is the first report on the OMT which can catalyze tri-methylation of stilbene and the first example of the microbial OMT which exhibits di-methylation activity toward stilbene. In case of flavonoids, an OMT from *Catharanthus roseus* (CrOMT2) can catalyze di-methylation of flavonol and flavanol (myricetin and dihydromyricetin) at the positions 3′ and 5′ [29]. The di-methylation of 6 and 3′ positions of 5,6,7,3′,4′-pentahydroxyflavonol (quercetagetin) by the OMT from *Mesembryanthemum crystallinum* (PFOMT) has also been reported [30]. To our knowledge, only one example of OMT showing tri-methylation activity exists to date, i.e., OMT from *Triticum aestivum* (TaOMT2) exhibits 3′,5′-di- and 3′,4′,5′-tri-methylation activities toward 5,7,3′,4′,5′-pentahydroxyflavone (tricetin) [31]. Thus, this is the first report of the microbial OMT that catalyzes the di- and tri-methylation of flavonoids.

By employing high methylation activity as well as the broad regiospecificity of SaOMT2, we successfully developed an *E. coli*–*Streptomyces* cocultivation system for carrying out microbial production of di- and tri-methylated flavonoids for the first time. The cocultivation of BL21/NRG and DHS2001/SaOMT2 produced 5-hydroxy-7,4′-dimethoxyflavanone, 4′-hydroxy-5,7-dimethoxyflavanone, and 5,7,4′-trimethoxyflavanone at titers of approximately 15 mg/L, 34 mg/L, and 11 mg/L, respectively (Fig. 6b). The coculture of BL21/APG with DHS2001/SaOMT2 produced approximately 24 mg/L of 5-hydroxy-7,4′-dimethoxyflavone and 1 mg/L of 5,7,4′-trimethoxyflavone (Fig. 6c). These are the first examples for the microbial production of multi-methylated flav(an)ones. High-yield production of tri-methylated stilbene was also achieved for the first time. A total titer of 29 mg/L of 3,5,4′-trimethoxystilbene was produced by cocultivating BL21/PTS with DHS2001/SaOMT2 (Fig. 6a). Previously, only 0.2 mg/L of this tri-methylated stilbene compound was produced by the *E. coli* strain expressing resveratrol biosynthetic genes together with two OMTs from *S. bicolor* [17]. Moreover, the SaOMT2-associated cocultivation system proved to be useful for the production of well-known mono-methylated flavonoids such as sakuranetin and genkwanin. In previous reports, approximately 40 mg/L of sakuranetin and genkwanin were produced by the engineered *E. coli* using tyrosine as substrate [15, 16]. In comparison to those previous reports, here, a higher level of sakuranetin (~ 79 mg/L) was produced along with a similar level of genkwanin (~ 42 mg/L) production from 4-coumaric acid (Fig. 6b, c).

Further optimization of culture conditions and metabolic engineering of both *E. coli* and *S. venezuelae* will improve the production levels of methylated phenylpropanoids. For instance, in the present study, the initial substrate, 4-coumaric acid, was fed at a concentration of 1.2 mM because this substrate concentration reportedly supports the production of phenylpropanoids in *S. venezuelae* without inhibiting the growth of the bacteria [19]. Additionally, the precultivation period for *E. coli* (3 h after induction) and *S. venezuelae* (40 h) was set in consideration of the different growth rates of these microbes before optimization of the mixing ratio. Therefore, further experimental optimization of the coculture conditions such as optimal substrate concentration and preculture period would improve the current titers. Metabolic engineering strategy such as the coexpression of SAM (a methyl donor) synthetase in the OMT-expressing *S. venezuelae* [31] as well as optimization of promoters and construct designs in the phenylpropanoid-producing *E. coli* [23] would also enhance the production levels. Finally, investigation of this multi-methylation activity of SaOMT2 toward more diverse phenylpropanoid substrates will expand the chemical diversity of this important class of natural products. Nevertheless, the cocultivation system involving phenylpropanoid-producing *E. coli* and SaOMT2-expressing *S. venezuelae* developed in this study proves to be an efficient biological tool for facilitating high-yield production of multi-methylated phenylpropanoid compounds.

## Conclusions

The previously unknown broad regiospecificity of OMT from *S. avermitilis* (SaOMT2) capable of multi-methylation of flavanone, flavone, and stilbene was discovered when SaOMT2 was expressed in *S. venezuelae*. The development of a cocultivation system based on phenylpropanoid-producing *E. coli* and SaOMT2-expressing *S. venezuelae* by exploiting this diverse regiospecificity of SaOMT2 resulted in efficient microbial production of di- and tri-methylated phenylpropanoids including 5-hydroxy-7,4′-dimethoxyflavanone, 4′-hydroxy-5,7-dimethoxyflavanone, 5,7,4′-trimethoxyflavanone, 5-hydroxy-7,4′-dimethoxyflavone, 5,7,4′-trimethoxyflavone, and 3,5,4′-trimethoxystilbene for the first time. Further optimization and application of this cocultivation strategy toward various phenylpropanoid compounds will facilitate the development of the abovementioned valuable compounds as pharmaceuticals and nutraceuticals.

## Methods

### Bacterial strains, plasmids, culture medium, and reagents

*Escherichia coli* DH5α was used for routine subcloning, whereas *E. coli* BL21(DE3) (Novagen, Madison, WI, USA) was used as a heterologous host for expressing the recombinant SaOMT2 and producing pterostilbene, naringenin, and apigenin. pGEM T-easy (Promega, Madison, WI, USA) vector was used for subcloning. The gene expression vectors for *E. coli* strain, namely pCDFduet-1 and pET32a, were purchased from Novagen (Madison, WI, USA). The pCDFduet-amp plasmid was prepared by inserting an ampicillin resistant marker for *E. coli*–*Streptomyces* coculture. *E. coli* strains were grown in LB medium [32] supplemented with suitable antibiotics. *S. avermitilis* ATCC 31267 was obtained from the American Type Culture Collection (Manassas, VA, USA). *S. venezuelae* DHS2001, in which the native pikromycin polyketide synthase gene was deleted [33], was used as a *Streptomyces* heterologous host. *S. venezuelae* mutants were grown in R2YE liquid medium [27] at 30 °C in a rotary shaker at 230 rpm. The high-copy number *E. coli*–*Streptomyces* shuttle vector pSE34 containing a strong constitutive ermE* promoter plus a thiostrepton resistance marker [21] was used as the expression vector in the *Streptomyces* heterologous host. For the expression of phenylpropanoid biosynthetic genes in an *E. coli* heterologous host, the Os4CL, VvSTS, PeCHS, MtCHI, PcFNS, VvROMT, and codon-optimized SaOMT2 genes were synthesized from Genotech (Daejon, Korea). All bacterial strains and plasmids used in this study are listed in Table 1.

Restriction endonucleases and T4 DNA ligase were purchased from New England BioLabs (Ipswich, MA, USA). The genomic DNA from *S. avermitilis* was isolated using a PureHelix™ Genomic DNA prep kit from NanoHelix (Daejon, Korea). PCR was performed using prime STAR^®^ GXL DNA polymerase from Takara (Kusatsu, Shiga, Japan). Ampicillin, thiostrepton, IPTG, SAM, 4-coumaric acid, resveratrol, pterostilbene, 3,5,4′-trimethoxystilbene, naringenin, sakuranetin, apigenin, genkwanin, 5,7,4′-trimethoxyflavone, and formic acid were obtained from Sigma-Aldrich (St Louis, MO, USA). Desoxyrhapontigenin (3,5-dihydroxy-4′-methoxystilbene), 5-hydroxy-3,4′-dimethoxystilbene, 5-hydroxy-7,4′-dimethoxyflavanone, 4′-hydroxy-5,7-dimethoxyflavanone, and 5,7,4′-trimethoxyflavanone were purchased from Carbosynth (Berkshire, UK). HPLC-grade acetonitrile (MeCN), ethyl acetate (EA), methanol (MeOH), and water were acquired from JT Baker (Phillipsburg, NJ, USA). All other chemicals were of the highest available purity grade.

### Construction of SaOMT2-expressing *S. venezuelae* mutant

For achieving heterologous expression of the SaOMT2 gene in *S. venezuelae*, a pSE-SaOMT2 plasmid was constructed. The SaOMT2 gene was amplified from the *S. avermitilis* genomic DNA using PCR and by employing primers SaOMT2-1_F/SaOMT2-1_R (Additional file 1: Table S1). A PCR-amplified DNA fragment was cloned into pGEM T-easy vector, and the digested SaOMT2 fragment was ligated into the complementary sites of pSE34 to generate pSE-SaOMT2 (Table 1). The expression plasmid pSE-SaOMT2 was transformed into *S. venezuelae* DHS2001, yielding the corresponding recombinant strain DHS2001/SaOMT2 (Table 1). *S. venezuelae* DHS2001 protoplast formation and transformation procedures were performed as described previously [27].

### Construction of the protein expression mutants for recombinant SaOMT2

The histidine-tagged recombinant SaOMT2 was expressed in both *S. venezuelae* and *E. coli* to characterize the methylation activity in vitro. For expression in *S. venezuelae*, PCR was performed using primers SaOMT2-1_F/SaOMT2-H1_R from *S. avermitilis* genomic DNA. The SaOMT2-H1_R primer sequence contained a six-histidine tag to facilitate the purification of the resulting recombinant protein (Additional file 1: Table S1). The PCR fragment was cloned into pGEM T-easy vector and subsequently ligated into the complementary sites of pSE34 to yield pSE-SaOMT2-H. The pSE-SaOMT2-H plasmid containing the SaOMT2 gene fused with C-terminal histidine-tag was transformed into *S. venezuelae* DHS2001, yielding the corresponding recombinant strain DHS2001/SaOMT2-H (Table 1).

To construct the pET-SaOMT2-H plasmid for expressing recombinant SaOMT2 in *E. coli*, the synthetic codon-optimized SaOMT2 gene was cloned into pGEM T-easy vector and ligated into the *Xba*I/*Not*I sites of pET32a to generate pET-SaOMT2-H. The pET-SaOMT2-H plasmid containing the SaOMT2 gene fused with C-terminal histidine-tag was transformed into *E. coli* BL21(DE3), yielding the corresponding recombinant strain BL21/SaOMT2-H (Table 1).

### Construction of phenylpropanoid-producing *E. coli* mutants

The pPTS plasmid containing Os4CL, VvSTS, and VvROMT genes was constructed for pterostilbene production. The restriction sites for the cloning of Os4CL, VvSTS, and VvROMT genes were introduced by PCR amplification using the primers Os4CL_F/Os4CL_R, VvSTS_F/VvSTS_R, and VvROMT_F/VvROMT_R, respectively (Additional file 1: Table S1). The PCR-amplified DNA fragments containing Os4CL, VvSTS, and VvROMT genes were cloned into pGEM T-easy vector, and the *Xba*I/*Not*I fragment carrying the entire combined DNA was transferred into the same sites of pET32a to generate pPTS. The expression plasmid, pPTS, was introduced into *E. coli* BL21(DE3), resulting in the formation of BL21/PTS (Table 1).

The pNRG plasmid containing PeCHS, Os4CL, and MtCHI genes was constructed to produce naringenin. The DNA fragments, containing the appropriate restriction sites to facilitate cloning, were prepared by PCR amplification using the primers PeCHS_F/PeCHS_R, Os4CL-2_F/Os4CL-2_R, and MtCHI_F/MtCHI_R and were subsequently cloned into pGEM T-easy vector, respectively (Additional file 1: Table S1). The entire combined DNA fragments containing PeCHS, Os4CL, and MtCHI genes were ligated into pCDFduet-amp using *Nde*I/*Avr*II, yielding the corresponding recombinant strain pNRG. For the production of methylated naringenin, the codon-optimized SaOMT2 gene, in which the restriction sites were introduced into the synthetic DNA fragment by performing PCR using primers SaOMT2-2_F/SaOMT2-2_R, was cloned into pGEM T-easy vector (Additional file 1: Table S1); the digested SaOMT2 fragment was ligated into the complementary sites of pNRG to generate pNRG-SaOMT2 (Additional file 1: Table S1). The expression plasmids, pNRG and pNRG-SaOMT2, were introduced into *E. coli* BL21(DE3), resulting in the formation of BL21/NRG and BL21/NRG-SaOMT2 (Table 1).

For producing apigenin in *E. coli* heterologous host, the pAPG plasmid containing PcFNS gene was constructed. The restriction sites were introduced in the synthetic PcFNS gene by performing PCR amplification using primers PcFNS_F/PcFNS_R (Additional file 1: Table S1). The PCR product was digested with *Kpn*I/*Avr*II and cloned into the complementary sites of pNRG to generate pAPG. Plasmid pAPG-SaOMT2 was constructed to produce the methylated apigenin. The *Avr*II/*Not*I-digested SaOMT2 fragment was ligated into the complementary sites of pAPG to generate pAPG-SaOMT2. The expression plasmids, pAPG and pAPG-SaOMT2, were introduced into *E. coli* BL21(DE3), thereby resulting in the formation of BL21/APG and BL21/APG-SaOMT2 (Table 1).

### In vivo bioconversion of SaOMT2 expressed in *S. venezuelae*

For in vivo bioconversion using the *S. venezuelae* expressing SaOMT2, the DHS2001/SaOMT2 strain was cultivated at 30 °C for 36 h in R2YE medium. 0.5 mL of seed cultures was inoculated into 50 mL of R2YE medium. After 48 h, the cultures were supplemented with 100 mM resveratrol, naringenin, or apigenin, which were subsequently incubated for an additional 40 h period prior to extraction.

### In vitro reaction using purified SaOMT2 expressed in *E. coli* and *S. venezuelae*

For the expression of recombinant SaOMT2 in *Streptomyces* host, 3 mL of 36 h-old cultivated DHS2001/SaOMT2-H were inoculated into 300 mL of fresh R2YE medium. Cells were grown at 30 °C for 48 h. Meanwhile, the BL21/SaOMT2-H strain was cultivated overnight in 10 mL of LB medium at 37 °C; subsequently, 4 mL of seed cultures were inoculated into 400 mL of LB medium [32]. The cells were cultured until an OD_600_ of 0.5–0.6, and the gene expression in the cell culture was induced with 1 mM IPTG. Simultaneously, the incubation temperature was shifted from 37 to 30 °C, and the cells were grown for an additional 6 h period.

The harvested cells were resuspended in Tris–HCl (pH 7.0) buffer solution and lysed by sonication using a VCX 500 from Sonics & Materials, Inc. (Newtown, CT, USA). The cell lysates were separated by centrifugation for 30 min at 4 °C, and then the supernatant was purified using Ni^2+^-NTA agarose chromatography columns. After washing the column with an NPI-40 buffer (300 mM NaCl, 40 mM imidazole, 50 mM sodium phosphate, pH 8.0), histidine-tagged recombinant proteins were eluted using NPI-500 buffer (300 mM NaCl, 500 mM imidazole, 50 mM sodium phosphate, pH 8.0). The purified proteins were analyzed by 12.5% SDS-PAGE and visualized using Coomassie brilliant blue. Typically, approximately 11 mg/L and 9 mg/L of purified SaOMT2 proteins were obtained from DHS2001/SaOMT2-H and BL21/SaOMT2-H, respectively.

The enzyme reaction involving purified SaOMT2 was carried out using a mixture comprising 25 mM Tris–HCl (pH 7.0) supplemented with 1.23 mM of purified SaOMT2, 100 mM apigenin, 100 μM MgCl_2_, and 100 μM SAM; this mixture was allowed to react for 12 h at 37 °C. The reaction was stopped by adding two volumes of EA and centrifuged at 13,000 rpm for 10 min. After the organic phase was evaporated to dryness, the residue was dissolved in MeOH. A portion of this solution was analyzed by UPLC–qTOF–HR-MS.

### Production of phenylpropanoid using *E. coli* mutants

The seed culture broth that was subjected to overnight incubation was subsequently inoculated into 30 mL LB medium at an OD_600_ value of 0.2 and cultivated until an OD_600_ of 0.7–0.8 was reached. At that point, the IPTG was added into the culture broth until the final concentration became 1 mM and continuously incubated at 30 °C for another 3 h. The cells were harvested at 4 °C by centrifugation (10 min at 6000 rpm) and resuspended with 15 mL of fresh R2YE medium. The culture was supplemented with 1.2 mM 4-coumaric acid plus 1 mM IPTG and further incubated for 18 h at 30 °C.

### Production of *O*-methylated phenylpropanoids using *E. coli*–*Streptomyces* cocultivation

For methylated phenylpropanoid production using *E. coli*–*Streptomyces* cocultivation, the seed cultures of *E. coli* mutants (BL21/PTS, BL21/NRG, or BL21/APG), which were subjected to overnight incubation, were inoculated into 100 mL LB medium at an OD_600_ value of 0.2. The culture broth was incubated at 37 °C until an OD_600_ of 0.7–0.8 was reached. Subsequently, the biosynthetic genes were induced with 1 mM IPTG, and the cells were grown at 30 °C for another 3 h. Meanwhile, 1 mL of 36 h-old cultivated DHS2001/SaOMT2 was inoculated into 100 mL of R2YE medium and grown at 30 °C for 48 h. *E. coli* and *Streptomyces* cells were mixed volumetrically according to the indicated inoculation ratios. The cocultures were fed with 1.2 mM of 4-coumaric acid plus 1 mM IPTG and allowed to grow for different periods at 30 °C.

### Isolation and structural analysis of flavanones and stilbenes

The cultures of *E. coli* and *S. venezuelae* mutants or their cocultures were extracted and partitioned twice using an equivalent volume of EA in a flask, and the EA layers of each culture were combined and concentrated in vacuo. Each EA extract was dissolved in MeOH and then analyzed by UPLC–qTOF–HR-MS.

### UPLC–qTOF–HR-MS analysis of flavonoid and stilbene analogs

UPLC–qTOF–HR-MS analysis of the flavonoid and stilbene analogs was performed on a Waters XEVO^®^ G2S Q-ToF mass spectrometer coupled with a Waters Acquity UPLC^®^ system equipped with an Xbridge^®^ C18 column (2.1 × 100 mm, 3.5 μm) consisting of an Acquity I-Class system. Gradient elution using solvent A (0.1% TFA) and solvent B (80% *aq*. MeCN with 0.1% TFA) as the mobile phase at a flow rate of 0.2 mL/min at 40 °C was performed. The MS system was operated in ESI with a positive ionization mode. The typical operating parameters were as follows: analyzer, resolution mode; capillary voltage (volt.), 3.0 kV; sampling cone volt., 30 V; source temperature, 120 °C; source offset, 80; desolvation temperature, 400 °C; cone gas flow, 10 L/h; desolvation gas flow, helium collision gas (600 L/h). The analyzer was operated with an extended dynamic range at 60,000 resolution (FWHM at *m/z* 556) and with an acquisition time of 0.1 s. Leucine enkephalin (400 pg/μL, 50% *aq*. MeCN with 0.1% formic acid) as a lockspray was infused at a rate of 5 μL/min for mass correction. Mass spectra were acquired over a scan range of 50–600 amu with a scan time 0.1 s.

## Additional file

**Additional file 1.** Figures S1–S5 and Table S1.

## Abbreviations

4CL: 4-coumarate:CoA ligase
CHI: chalcone isomerase
CHS: chalcone synthase
FNS: flavone synthase
MtCHI: CHI from *Medicago truncatula*
OMT: *O*-methyltransferase
Os4CL: 4CL from *Oryza sativa*
PcFNS: FNS from *Petroselinum crispum*
PeCHS: CHS from *Populus euramericana*
qTOF-HR-MS: quadrupole time-of-flight high resolution mass spectrometry
SaOMT2: OMT from *Streptomyces avermitilis*
STS: stilbene synthase
UPLC: ultra performance liquid chromatography
VvROMT: OMT from *Vitis vinifera*
VvSTS: STS from *Vitis vinifera*
