# Supplementary material for: High-yield production of multiple O-methylated phenylpropanoids by the engineered Escherichia coli–Streptomyces cocultivation system
Source: Microb Cell Fact. 2019 Apr 10;18:67. doi: 10.1186/s12934-019-1118-9 (PMC6456975; doi:10.1186/s12934-019-1118-9)
Supplement: Supplementary file 1 — Additional file 1. Figures S1–S5 and Table S1. [file 12934_2019_1118_MOESM1_ESM.pdf]

Additional file

High-yield production of multiple *O*-methylated phenylpropanoids by the engineered *Escherichia coli-Streptomyces* cocultivation system

Heqing Cui[1†], Myoung Chong Song[1†], Yeon Hee Ban[1], Soo Youn Jun[2], An Sung Kwon[2], Ji Young Lee[1] and Yeo Joon Yoon[1*]

*Correspondence: joonyoon@ewha.ac.kr

[†]Equal contributors

[1]Department of Chemistry and Nanoscience, Ewha Womans University, Ewhayeodae-gil 52, Seoul, 03760, Republic of Korea

[2]iNtRON Biotechnology, Inc., Seongnam-si, Gyeonggi-do 13202, Republic of Korea

Email addresses:

Heqing Cui (cuiheqing810@hotmail.com); Myoung Chong Song (smch517@ewha.ac.kr);

Yeon Hee Ban (yhban@ewha.ac.kr); Soo Youn Jun (jsy@intron.co.kr);

An Sung Kwon (kwon4053@intron.co.kr); Ji Young Lee (jji123789@naver.com);

Yeo Joon Yoon (joonyoon@ewha.ac.kr)

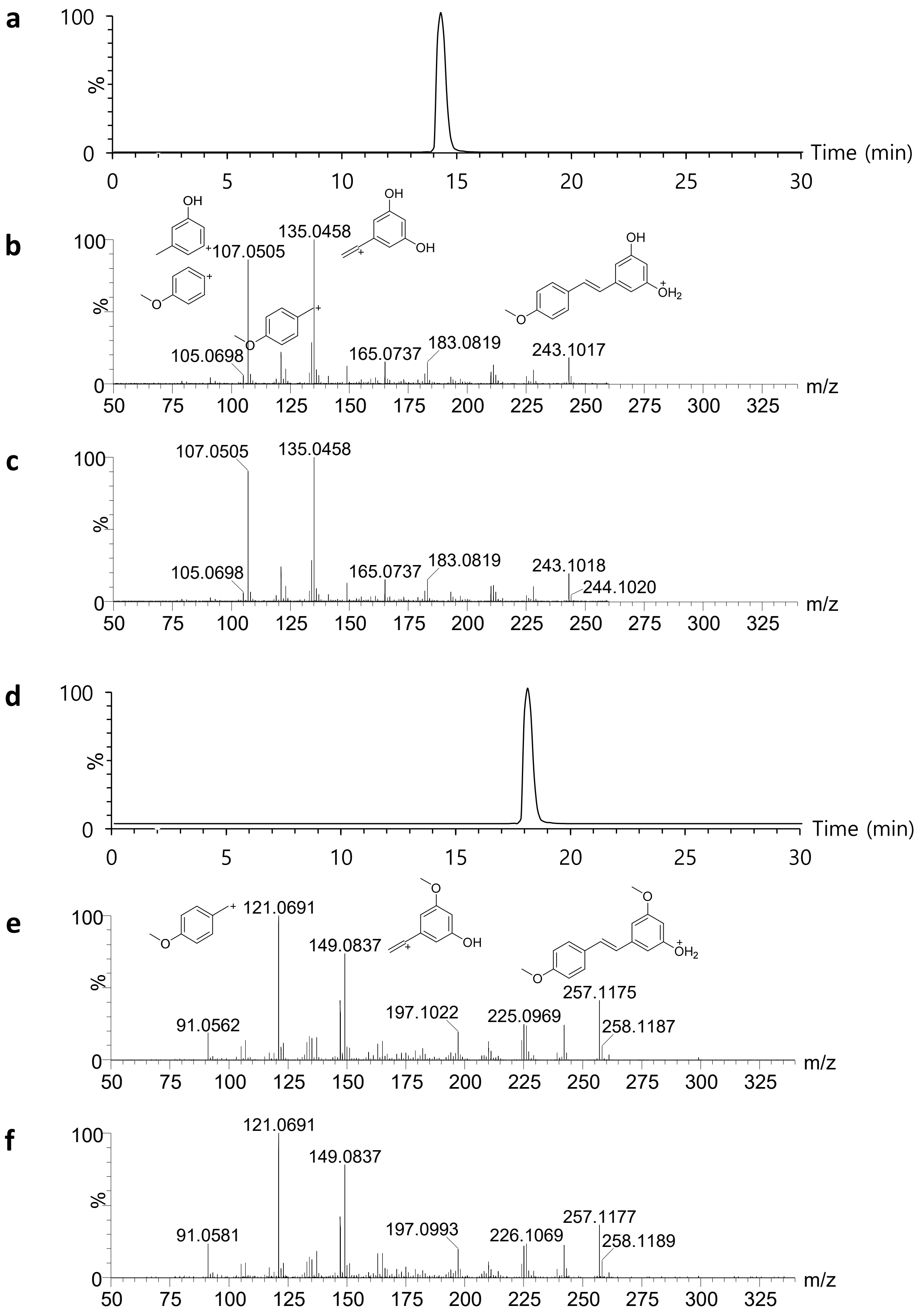
a
100
%
0
Time (min)
0 5 10 15 20 25 30
b
107.0505
135.0458
105.0698
165.0737
183.0819
243.1017
m/z
50 75 100 125 150 175 200 225 250 275 300 325
c
107.0505
135.0458
105.0698
165.0737
183.0819
243.1018
244.1020
d
Time (min)
e
121.0691
149.0837
91.0562
197.1022
225.0969
257.1175
258.1187
f
121.0691
149.0837
91.0581
197.0993
226.1069
257.1177
258.1189

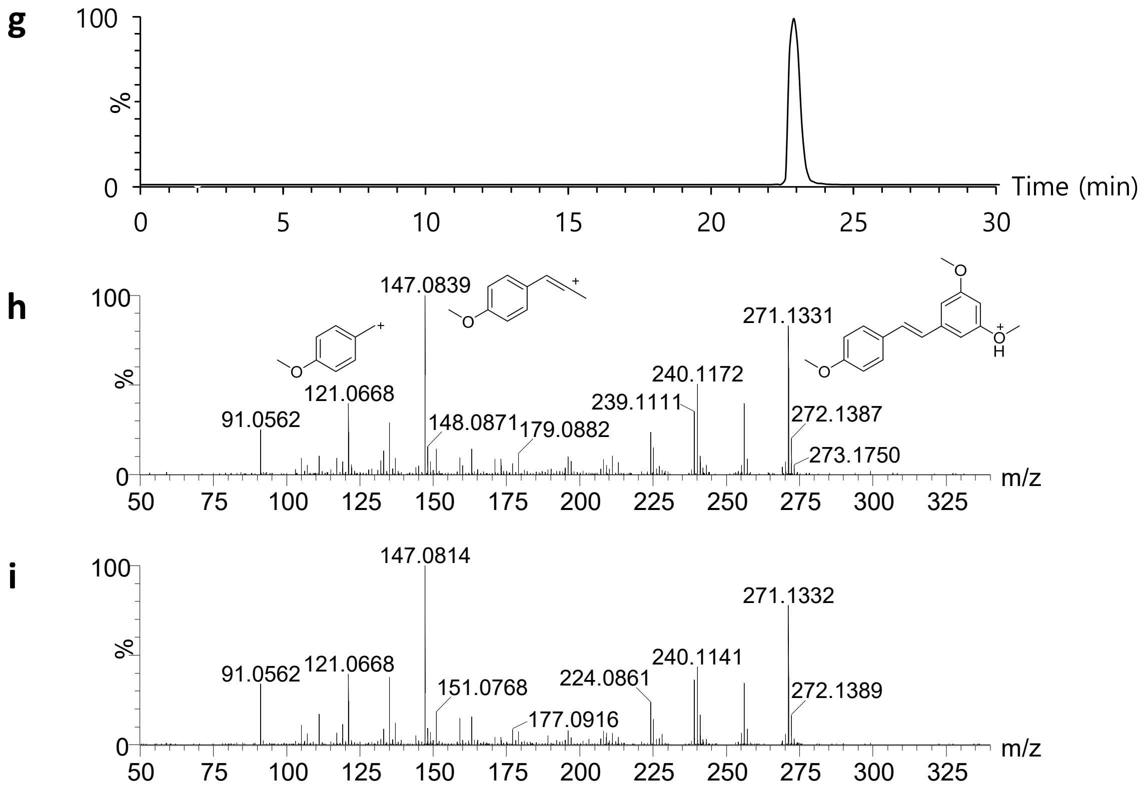

Figure S1. UPLC–qTOF–HR-MS analyses of the methylated stilbenes produced by *S. venezuelae* DHS2001 harboring pSE-SaOMT2 (DHS2001/SaOMT2). (**a**) UPLC–qTOF–HR-MS chromatogram selected for *m/z*=243.1016 corresponding to standard desoxyrhapontigenin. (**b**) MS/MS spectrum of standard desoxyrhapontigenin. (**c**) MS/MS spectrum of desoxyrhapontigenin from the culture extracts of DHS2001/SaOMT2 supplemented with resveratrol. (**d**) UPLC–qTOF–HR-MS chromatogram selected for *m/z*=257.1172 corresponding to standard 5-hydroxy-3,4′-dimethoxystilbene. (**e**) MS/MS spectrum of standard 5-hydroxy-3,4′-dimethoxystilbene. (**f**) MS/MS spectrum of 5-hydroxy-3,4′-dimethoxystilbene from the culture extracts of DHS2001/SaOMT2 supplemented with resveratrol. (**g**) UPLC–qTOF–HR-MS chromatogram selected for *m/z*=271.1329 corresponding to standard 3,5,4′-trimethoxystilbene. (**h**) MS/MS spectrum of standard 3,5,4′-trimethoxystilbene. (**i**) MS/MS spectrum of 3,5,4′-trimethoxystilbene from the culture extracts of DHS2001/SaOMT2 supplemented with resveratrol.

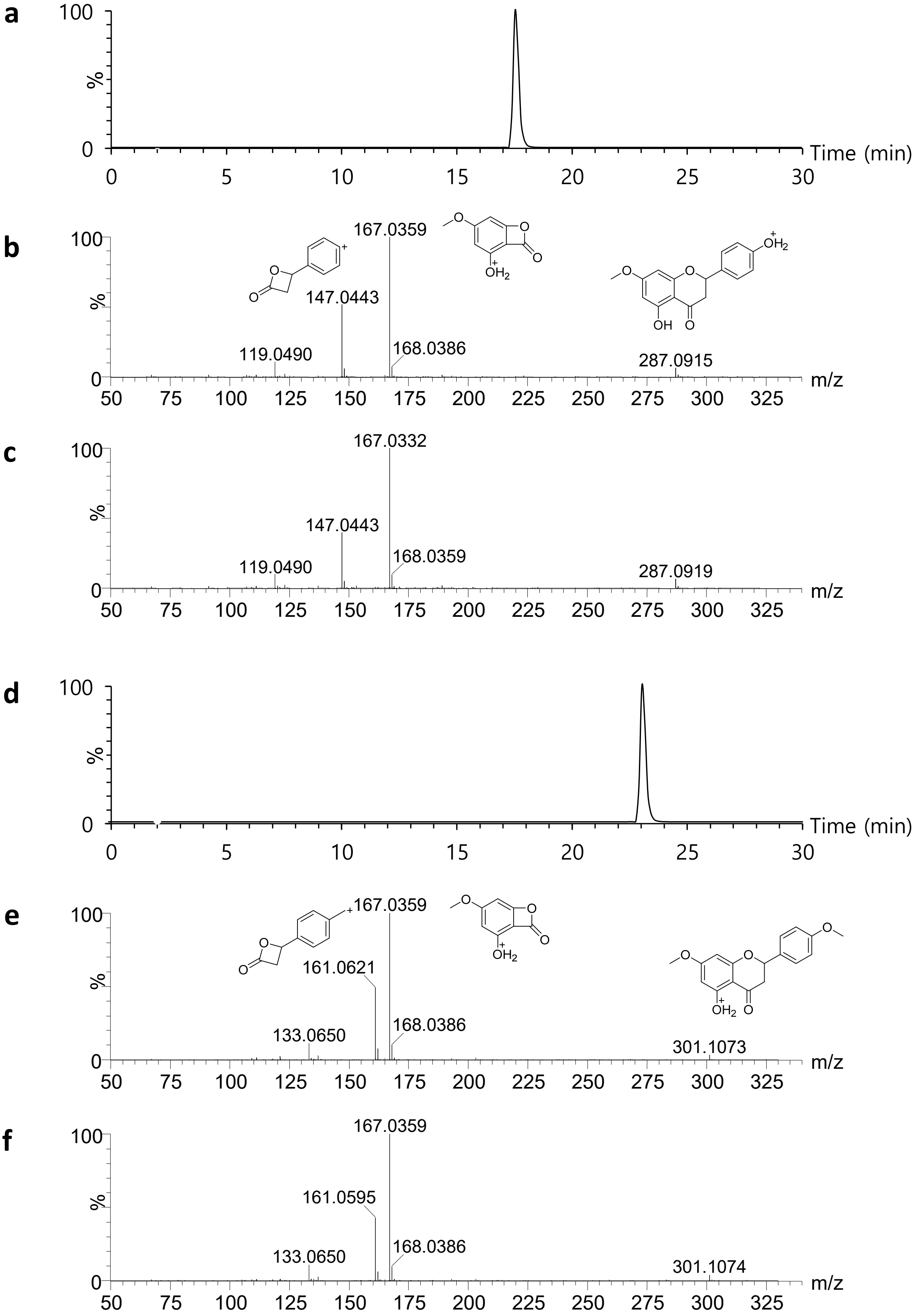

a
100
%
0
Time (min)
0
5
10
15
20
25
30
b
167.0359
147.0443
119.0490
168.0386
287.0915
m/z
c
167.0332
147.0443
119.0490
168.0359
287.0919
m/z
d
Time (min)
e
167.0359
161.0621
133.0650
168.0386
301.1073
m/z
f
167.0359
161.0595
133.0650
168.0386
301.1074
m/z
50
75
100
125
150
175
200
225
250
275
300
325

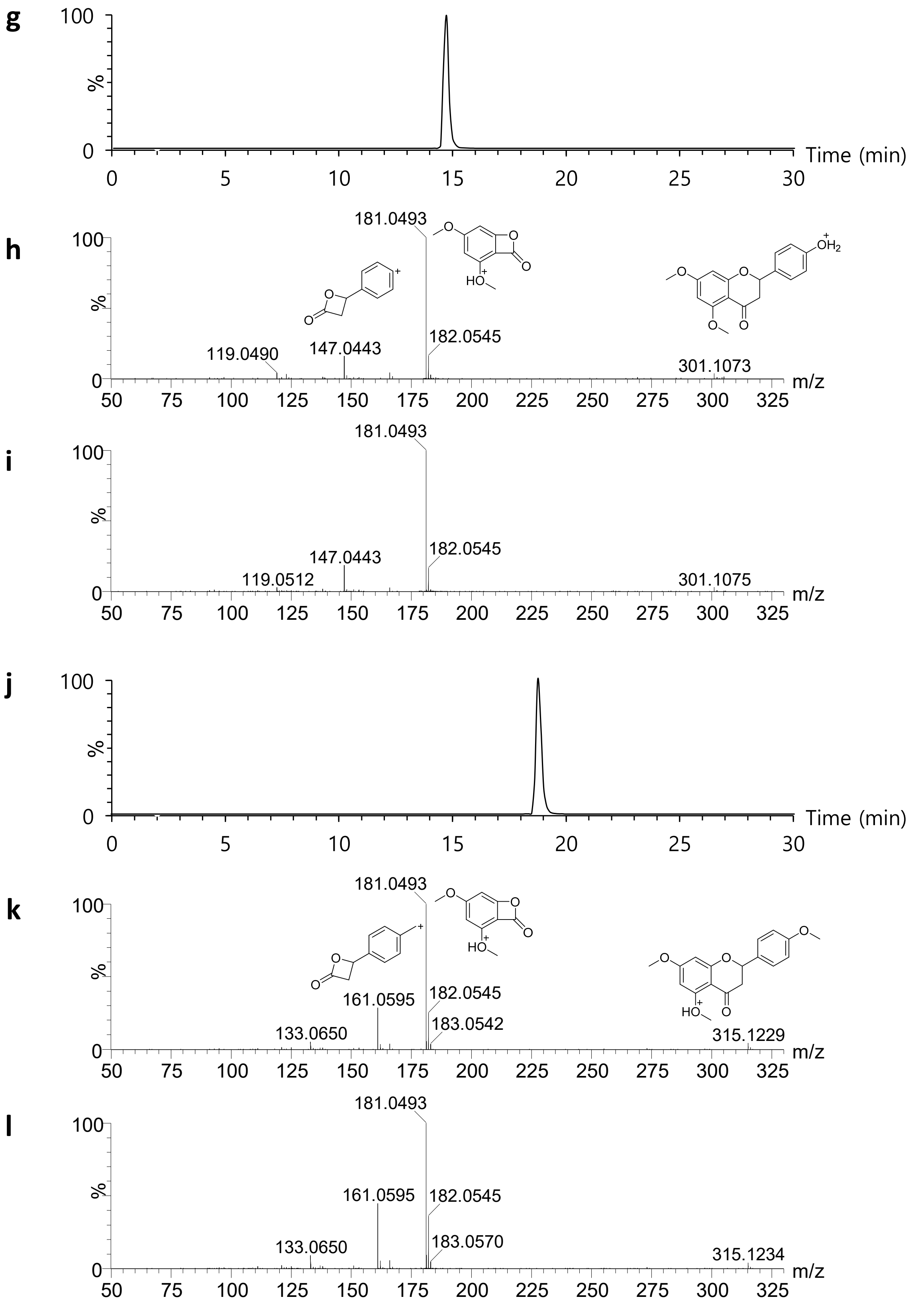

Figure S2. UPLC–qTOF–HR-MS analyses of the methylated flavanones produced by *S. venezuelae*

DHS2001 harboring pSE-SaOMT2 (DHS2001/SaOMT2). (**a**) UPLC-qTOF-MS chromatogram selected for *m/z*=287.0914 corresponding to standard sakuranetin. (**b**) MS/MS spectrum of standard sakuranetin. (**c**) MS/MS spectrum of sakuranetin from the culture extracts of DHS2001/SaOMT2 supplemented with naringenin. (**d**) UPLC–qTOF–HR-MS chromatogram selected for *m/z*=301.1071 corresponding to standard 5-hydroxy-7,4′-dimethoxyflavanone. (**e**) MS/MS spectrum of standard 5-hydroxy-7,4′-dimethoxyflavanone. (**f**) MS/MS spectrum of 5-hydroxy-7,4′-dimethoxyflavanone from the culture extracts of DHS2001/SaOMT2 supplemented with naringenin. (**g**) UPLC–qTOF–HR-MS chromatogram selected for *m/z*=301.1071 corresponding to standard 4′-hydroxy-5,7-dimethoxyflavanone. (**h**) MS/MS spectrum of standard 4′-hydroxy-5,7-dimethoxyflavanone. (**i**) MS/MS spectrum of 4′-hydroxy-5,7-dimethoxyflavanone from the culture extracts of DHS2001/SaOMT2 supplemented with naringenin. (**j**) UPLC–qTOF–HR-MS chromatogram selected for *m/z*=315.1227 corresponding to standard 5,7,4′-trimethoxyflavanone. (**k**) MS/MS spectrum of standard 5,7,4′-trimethoxyflavanone. (**l**) MS/MS spectrum of 5,7,4′-trimethoxyflavanone from the culture extracts of DHS2001/SaOMT2 supplemented with naringenin.

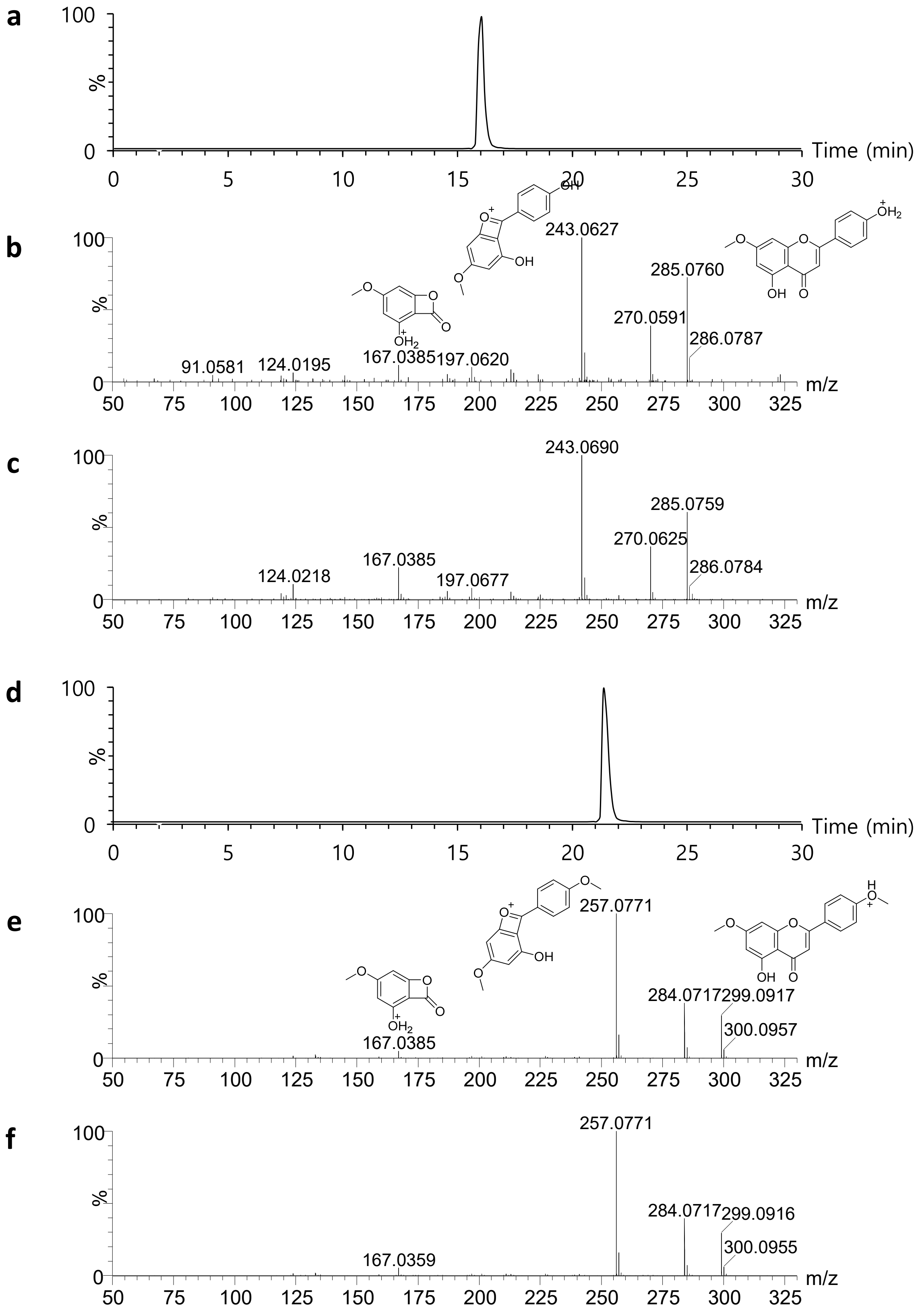
a
100
%
0
Time (min)
0 5 10 15 20 25 30
b
100
%
0
243.0627
285.0760
270.0591
286.0787
91.0581
124.0195
167.0385
197.0620
m/z
50 75 100 125 150 175 200 225 250 275 300 325
OH
OH
O
O
OH₂
O
OH
c
100
%
0
243.0690
285.0759
270.0625
286.0784
124.0218
167.0385
197.0677
m/z
50 75 100 125 150 175 200 225 250 275 300 325
d
100
%
0
Time (min)
0 5 10 15 20 25 30
e
100
%
0
257.0771
284.0717
299.0917
300.0957
167.0385
m/z
50 75 100 125 150 175 200 225 250 275 300 325
OH
OH
O
H
f
100
%
0
257.0771
284.0717
299.0916
300.0955
167.0359
m/z
50 75 100 125 150 175 200 225 250 275 300 325

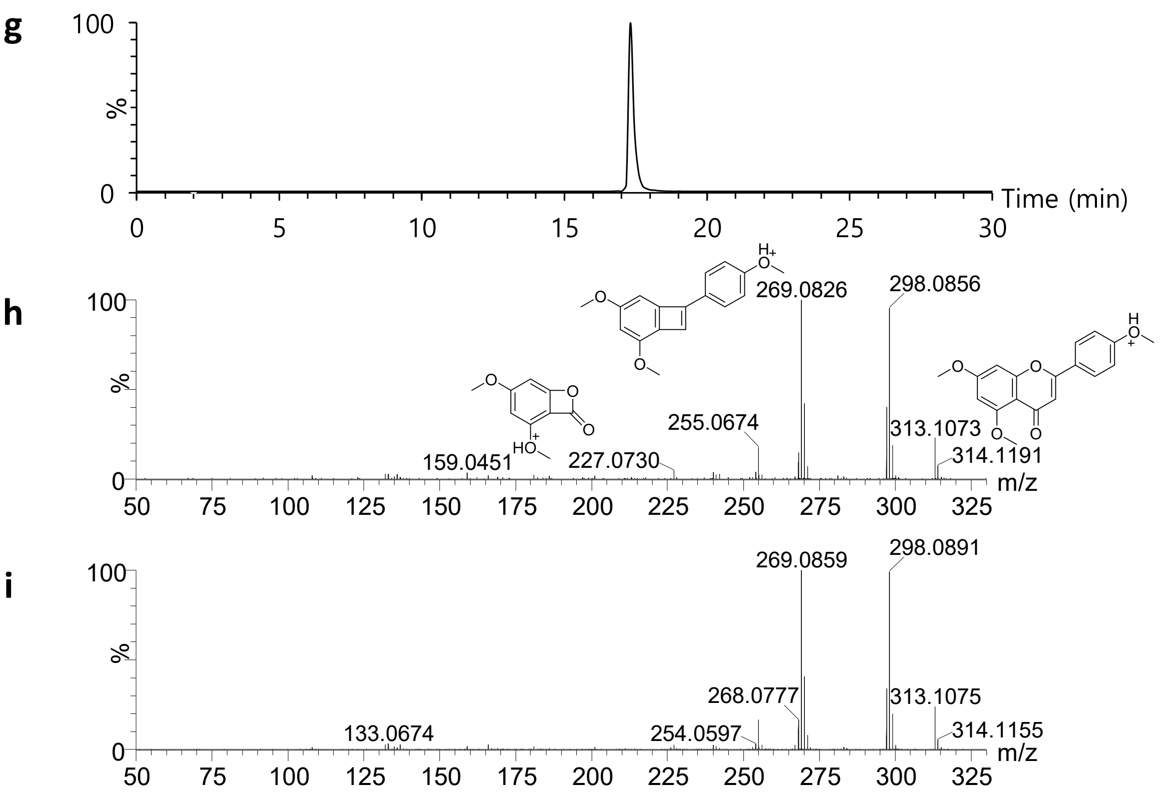

Figure S3. UPLC–qTOF–HR-MS analyses of the methylated flavones produced by *S. venezuelae* DHS2001 harboring pSE-SaOMT2 (DHS2001/SaOMT2). (**a**) UPLC–qTOF–HR-MS chromatogram selected for *m/z*=285.0757 corresponding to standard genkwanin. (**b**) MS/MS spectrum of standard genkwanin. (**c**) MS/MS spectrum of genkwanin from the culture extracts of DHS2001/SaOMT2 supplemented with apigenin. (**d**) UPLC–qTOF–HR-MS chromatogram selected for *m/z*=299.0914 corresponding to standard 5-hydroxy-7,4′-dimethoxyflavone. (**e**) MS/MS spectrum of standard 5-hydroxy-7,4′-dimethoxyflavone. (**f**) MS/MS spectrum of 5-hydroxy-7,4′-dimethoxyflavone from the culture extracts of DHS2001/SaOMT2 supplemented with apigenin. (**g**) UPLC–qTOF–HR-MS chromatogram selected for *m/z*=313.1071 corresponding to standard 5,7,4′-trimethoxyflavone. (**h**) MS/MS spectrum of standard 5,7,4′-trimethoxyflavone. (**i**) MS/MS spectrum of 5,7,4′-trimethoxyflavone from the culture extracts of DHS2001/SaOMT2 supplemented with apigenin.

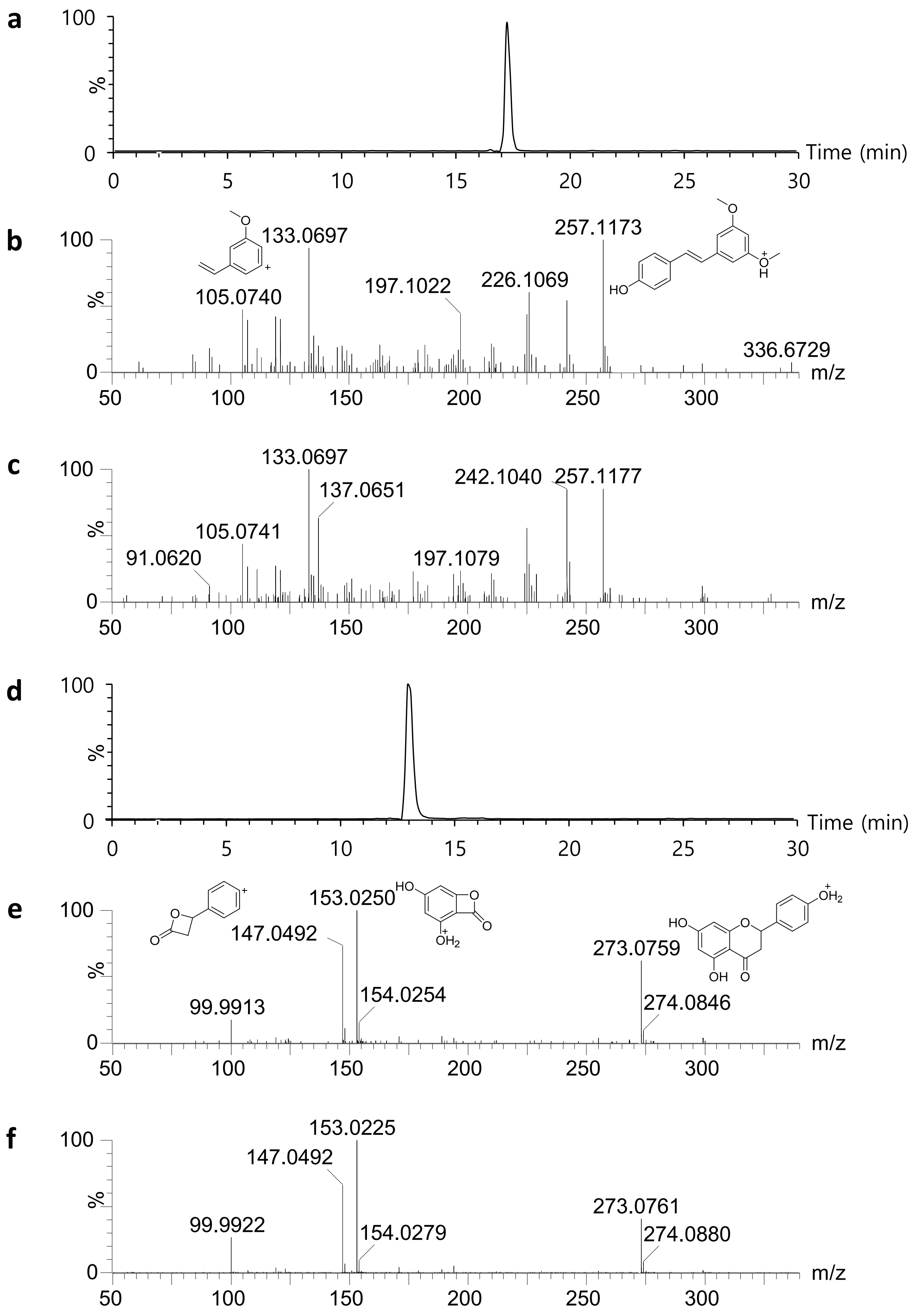
a
100
%
0
Time (min)
0
5
10
15
20
25
30
b
133.0697
105.0740
197.1022
226.1069
257.1173
HO
336.6729
m/z
50
100
150
200
250
300
c
133.0697
137.0651
242.1040
257.1177
105.0741
91.0620
197.1079
d
e
153.0250
147.0492
154.0254
99.9913
273.0759
274.0846
HO
OH
f
153.0225
147.0492
154.0279
99.9922
273.0761
274.0880

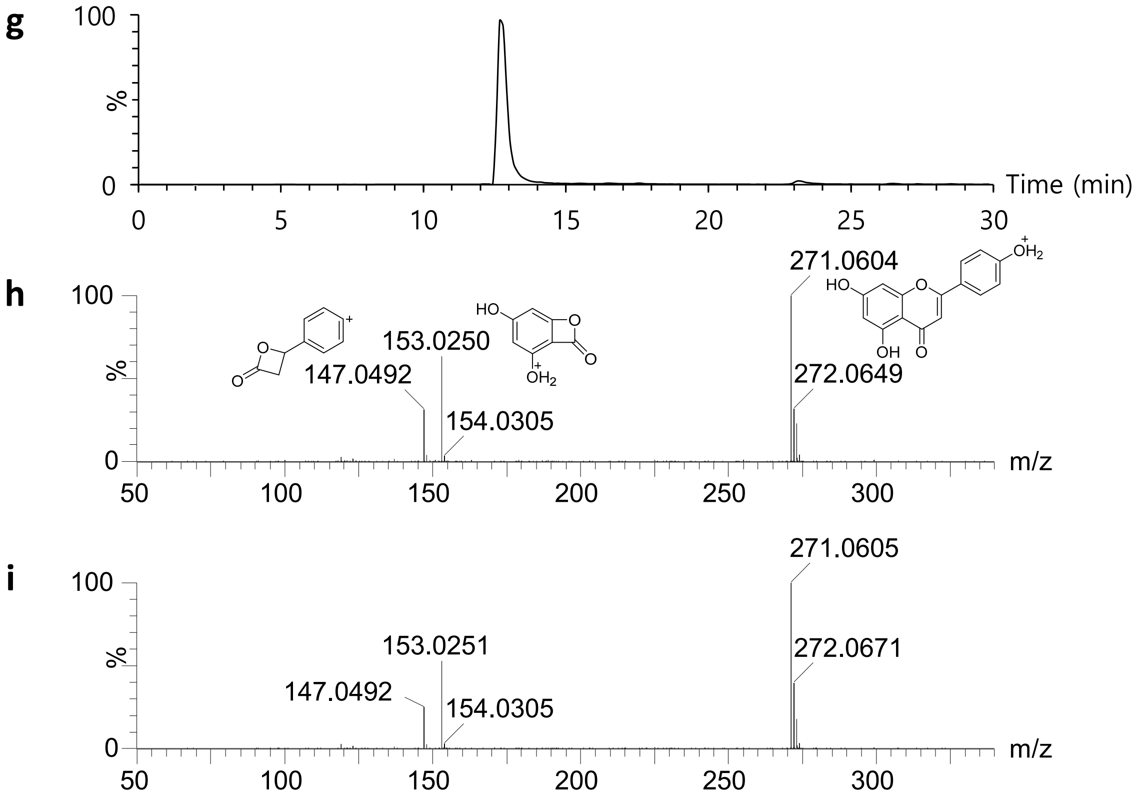

Figure S4. UPLC–qTOF–HR-MS analyses of the pterostilbene, naringenin and apigenin produced by *E. coli* mutants. (**a**) UPLC–qTOF–HR-MS chromatogram selected for *m/z*=257.1172 corresponding to standard pterostilbene. (**b**) MS/MS spectrum of standard pterostilbene. (**c**) MS/MS spectrum of pterostilbene obtained from BL21/PTS supplemented with 4-coumaric acid. (**d**) UPLC–qTOF–HR-MS chromatogram selected for *m/z*=273.0757 corresponding to standard naringenin. (**e**) MS/MS spectrum of standard naringenin. (**f**) MS/MS spectrum of naringenin obtained from BL21/NRG supplemented with 4-coumaric acid. (**g**) UPLC–qTOF–HR-MS chromatogram selected for *m/z*=271.0601 corresponding to standard apigenin. (**h**) MS/MS spectrum of standard apigenin. (**i**) MS/MS spectrum of apigenin obtained from BL21/APG supplemented with 4-coumaric acid.

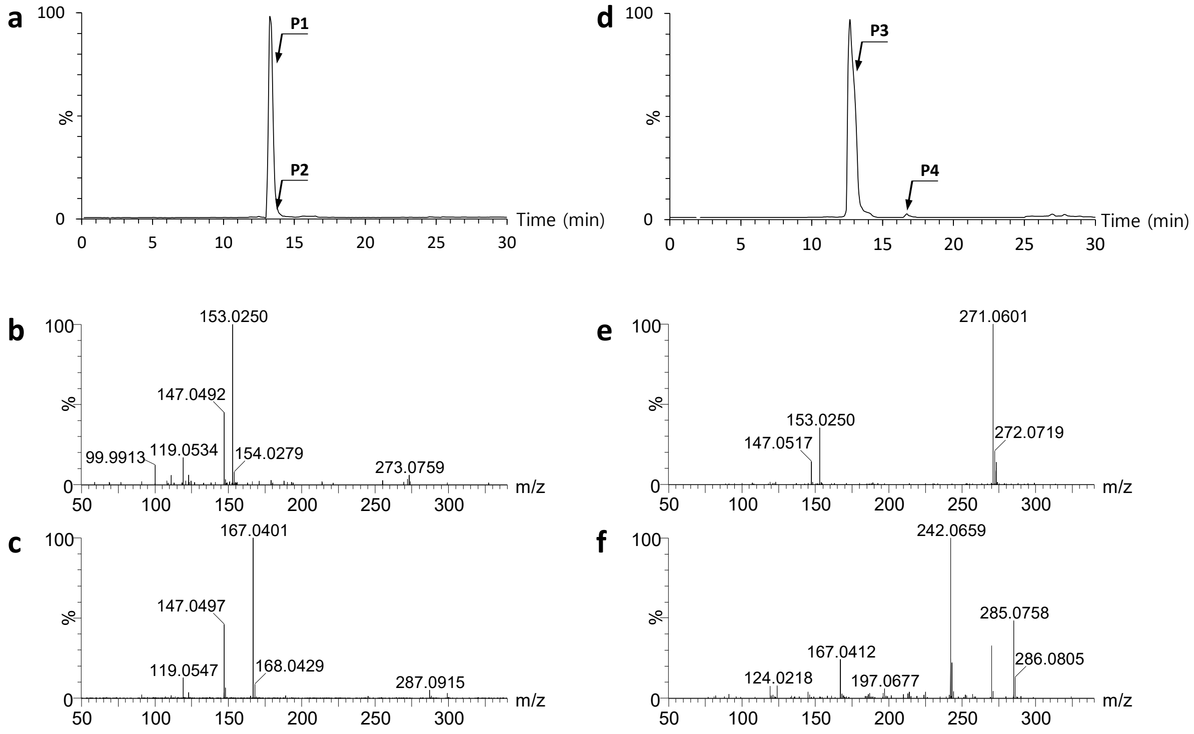

Figure S5. UPLC–qTOF–HR-MS analyses of methylated flavanone and methylated flavone produced by *E. coli* BL21(DE3) harboring pNRG-SaOMT2 and pAPG-SaOMT2, respectively. (**a**) UPLC–qTOF–HR-MS chromatogram selected for *m/z*=273.0757 and 287.0914 corresponding to naringenin (**P1**) and sakuranetin (**P2**), respectively, of culture extracts from BL21/NRG-SaOMT2. (**b**) MS/MS spectrum selected for *m/z*=273.0757 of naringenin obtained from BL21/NPG-SaOMT2 supplemented with 4-coumaric acid. (**c**) MS/MS spectrum selected for *m/z*=287.0914 of sakuranetin obtained from BL21/NPG-SaOMT2 supplemented with 4-coumaric acid. (**d**) UPLC–qTOF–HR-MS chromatogram selected for *m/z*=271.0601 and 285.0757 corresponding to apigenin (**P3**) and genkwanin (**P4**), respectively, of culture extracts obtained from BL21/APG-SaOMT2. (**e**) MS/MS spectrum selected for *m/z*=271.0601 of apigenin obtained from BL21/APG-SaOMT2 supplemented with 4-coumaric acid. (**f**) MS/MS spectrum selected for *m/z*=285.0757 of genkwanin obtained from BL21/APG-SaOMT2 supplemented with 4-coumaric acid.

Table S1. List of oligonucleotide primers used in this study

**Primers**	**Primer sequences (5′ to 3′,** restriction site underlined)
SaOMT2-1_F	GAATTCATGAGCTGCCGCACCGGC
SaOMT2-1_R	CCTAGGTCAACCTACGGCCCGCAA
SaOMT2-H1_R	CCTAGGTCAGTGGTGGTGGTGGTGGTGGCCAACCGCGCGCAGTTC
SaOMT2-H2_F	TCTAGAAATAATTTTGTTTAACTTTAAGAAGGAGATATACATATGAGTTGT CGTACTGGT
SaOMT2-H2_R	GCGGCCGCACCTACGGCCCGCAA
Os4CL_F	TCTAGAGGAGGATTACAATGGGGTCGGTGGCGGCG
Os4CL_R	AAGCTTTTAGCTGCTTTTGGGCGC
VvSTS_F	AAGCTTAGGAGGATTACAAAATGGCTTCAGTCGAGGAA
VvSTS_R	CCTAGGTTAATTTGTAACCAT
VvROMT_F	CCTAGGAGGAGGATTACAAAATGGATTTGGCAAACGGT
VvROMT_R	GCGGCCGCTCAAGGATAAACCTCAAT
Os4CL-2_F	AAGCTTAGGAGGATTACAATGGGGTCGGTGGCGGCG
Os4CL-2_R	GCGGCCGCTTAGCTGCTTTTGGGCGC
PeCHS_F	CCATGGAGATGGCACCGTCGATTGAG
PeCHS_R	AAGCTTATAAGCTTTCATGAGTAAATTGTTTG
MtCHI_F	CATATGATGGCTGCATCAATCACC
MtCHI_R	CCTAGGGGTACCTCAGTTTCCAATCTTGAA
PcFNS_F	GGTACCAGGAGGATTACAATGGCTCCTACAACAATA
PcFNS_R	CCTAGGCTAAGCTAAATTTTCATC
SaOMT2-2_F	CCTAGGAGGAGGTCTCGCATGAGTTGTCGTACTGGT
SaOMT2-2_R	GCGGCCGCTCAACCTACGGCCCGCAA
